# Helmet Continuous Positive Airway Pressure for Acute Bronchiolitis Respiratory Failure in a Pediatric Ward: Is It a Replicable Experience?

**DOI:** 10.3390/children11111273

**Published:** 2024-10-22

**Authors:** Anna Maria Musolino, Sabrina Persia, Maria Chiara Supino, Francesca Stoppa, Lelia Rotondi Aufiero, Raffaella Nacca, Laura Papini, Mara Pisani, Sebastian Cristaldi, Anna Chiara Vittucci, Livia Antilici, Corrado Cecchetti, Massimiliano Raponi, Vinay Nadkarni, Alberto Villani

**Affiliations:** 1 Emergency, Acceptance and General Pediatrics Department, Bambino Gesù Children’s Hospital IRCCS, Piazza Sant’Onofrio 4, 00165 Rome, Italy; sabrina.persia@opbg.net (S.P.); mariachiara.supino@opbg.net (M.C.S.); francesca.stoppa@opbg.net (F.S.); lelia.rotondiaufiero@opbg.net (L.R.A.); raffaella.nacca@opbg.net (R.N.); laura.papini@opbg.net (L.P.); mara.pisani@opbg.net (M.P.); sebastian.cristaldi@opbg.net (S.C.); annachiara.vittucci@opbg.net (A.C.V.); livia.antilici@opbg.net (L.A.); corrado.cecchetti@opbg.net (C.C.); alberto.villani@opbg.net (A.V.); 2 Bambino Gesù Children’s Hospital IRCCS, 00165 Rome, Italy; massimiliano.raponi@opbg.net; 3 Department of Anesthesiology and Critical Care Medicine, Children’s Hospital of Philadelphia, University of Pennsylvania Perelman School of Medicine, Philadephia, PA 19104, USA; nadkarni@chop.edu; 4 System Medicine Department, Tor Vergata University of Rome, 00133 Rome, Italy

**Keywords:** bronchiolitis, respiratory failure, Helmet Continuous Positive Airway Pressure (H-CPAP), infant, pediatric ward

## Abstract

(1) Background: Helmet Continuous Positive Airway Pressure (H-CPAP) has primarily been used in intensive care settings to treat moderate-to-severe bronchiolitis in infants. We aim to report on the feasibility of H-CPAP for selected infants with bronchiolitis in a pediatric ward. (2) Methods: A retrospective, observational, consecutive case series was studied of 26 patients who received H-CPAP on the pediatric ward from October 2022 to February 2023, including a description of patient outcomes and costs. (3) Results: Of 130 infants with bronchiolitis admitted to Bambino Gesù Hospital in Rome, 34 were hospitalized for moderate to severe bronchiolitis, and 26 began H-CPAP on the ward. Among the 26 pediatric patients who received H-CPAP on the ward, 4 out of 26 (15%) required transfer to the PICU within the first hours of care due to clinical deterioration. No problems with the H-CPAP interface or side effects attributable to H-CPAP were reported. Pharmacological sedation with a single dose of dexmedetomidine was required for 15/26 patients (57%) following failure of non-pharmacological anxiety reduction strategies. After introducing H-CPAP in our pediatric ward, we achieved total cost savings of approximately EUR 147,120. (4) Conclusions: Treatment with H-CPAP for infants with bronchiolitis may be feasible in non-intensive care settings with trained staff, appropriate monitoring, and rapid access to pediatric intensive care.

## 1. Introduction

Acute bronchiolitis is a leading cause of respiratory failure and the need for mechanical ventilatory support in hospitalized infants [1]. Globally, 34 million new cases of lower respiratory infections caused by respiratory syncytial virus occur in children under five years, leading to 3.4 million hospital admissions and nearly 199,000 deaths, annually [2]. Supportive treatment options include oxygen therapy, a high-flow nasal cannula (HFNC) [3,4], continuous positive airway pressure (CPAP) [5,6], bi-level positive airway pressure (BiPAP), invasive mechanical support, and ECMO. CPAP, widely used in pediatric intensive care units (PICUs), can reduce infants’ work when breathing [7], duration of mechanical ventilation, and length of hospital stay. Respiratory failure associated with bronchiolitis exhibits a seasonal pattern, and PICUs are often overwhelmed during peaks in incidence [8]. Building upon adult [6,9,10] and limited pediatric [11] H-CPAP experience that emerged during the COVID-19 pandemic, we began implementing H-CPAP for patients with moderate to severe acute bronchiolitis in a pediatric ward. Although this approach has been reported as feasible in selected general pediatric ward settings [12,13], we describe our experience and clinical outcomes with a Helmet-CPAP strategy initiated in a pediatric ward (PW) setting in Italy.

## 2. Materials and Methods

This retrospective descriptive, cohort, observational study was performed at Bambino Gesù Children’s Hospital, a tertiary care academic hospital in Rome, Italy. The study protocol was approved by the Institutional Review Board and Ethics Committee of the institution (Protocol 3305_OPBG_2023 approved on 27 May 2024). This study was conducted in line with the principles of the Declaration of Helsinki and its later amendments or comparable ethical standards. Informed consent was obtained retrospectively from all parents/legal guardians of the children after this study was approved by the ethics committee.

### 2.1. Respiratory Assistance Protocol

We managed respiratory assistance according to our hospital clinical pathway [14] based upon contemporary guidelines [7] for the management of acute respiratory failure in bronchiolitis. Oxygen supplementation was initiated when SpO_2_ values fell below 90–92% using nasal cannulas or a nasal/oral mask, aiming to maintain SpO_2_ levels above 93–94%. The transition to high-flow nasal cannula oxygen therapy occurred when PaCO_2_ was >50 mmHg or SpO_2_ < 90% persisted (in patients without chronic cardiac or respiratory conditions), accompanied by increased work when breathing and a respiratory rate > 50 breaths/min.

The indications for starting H-CPAP on the ward were as follows:

(1) As a rescue therapy for patients initially treated with HFNC or nasal cannula oxygen therapy who had SpO_2_ < 92% with FiO_2_ of 0.6, in the absence of hypercapnic respiratory failure and/or a critical increase in work when breathing.
(2) If one or more of the following were detected at the first evaluation of the patient:
  - Signs of respiratory fatigue with moderate/severe dyspnea (respiratory rate > 50 breaths/min);
  - Use of accessory respiratory muscles and paradoxical abdominal movement;
  - Hypoxemia despite high FiO_2_ (P/F ≤ 250) ± mild hypercapnia (up to 50 mmHg in arterial blood gas).

Helmet-CPAP failure was prospectively defined based on a clinician’s determination of the need for orotracheal intubation, transfer to the pediatric intensive care unit (PICU), or death. A lack of clinical improvement in the first 4 h of H-CPAP therapy, worsening respiratory distress, deterioration of blood gas exchange, or an inability to tolerate the interface were considered key indicators for transfer to the PICU. The decision on the best clinical setting for the patient was made collaboratively between the pediatric ward and consultative PICU teams, with situational awareness of the potential for rapid admission to the PICU in the event of clinical deterioration. The communication process was mandatory, brief, and facilitated by the proximity of the PICU to our pediatric ward (located on the same floor, less than 100 m apart).

### 2.2. H-CPAP Management

#### 2.2.1. H-CPAP Equipment

The Helmet-CPAP device used in our pediatric ward (PW) has the following components:

- A transparent, latex-free polyvinyl chloride helmet;
- An anti-asphyxia valve positioned on the outside of the head hood, allowing for manual opening if the source of gas flow malfunctions, or automatic activation if the pressure inside the helmet drops to <3 cmH_2_O;
- An expiratory limb;
- An inspiratory limb;
- An audio silencer to reduce noise inside the helmet by approximately 10 Db;
- Two sealed accesses for probes and catheters of 3.5–7.0 mm;
- An airtight patient access porthole with screw closure;
- A PEEP valve.

The device model we used was a Starmed Castar (Intersurgical^®^, Wokingham, UK). To ensure the sealing effect was tight but comfortable, the size of the helmet was chosen on the basis of the patient’s body weight. Three sizes (small, medium, and large) were available. An air–oxygen blender device allowed for a titrated flow rate, PEEP, and FiO_2_, with the flow rate always maintained >30 L/m to minimize the risk of CO_2_ rebreathing.

We did not use a humidifier due to the risk of condensation. 

#### 2.2.2. H-CPAP Procedure and Monitoring

Our pediatric ward had 12 beds available for admission. The nursing staff on the ward was organized as follows: 2 nurses and 1 nurse assistant on the morning and evening shifts, 2 nurses on the night shift. Four H-CPAP machines were available simultaneously. One parent was permitted to be present 24 h a day.

The setup procedure involved the following:

- Positioning the infant at a 45° angle using an oxygen nasal cannula or HFNC until the H-CPAP circuit was fully assembled;
- Connecting the FiO_2_ analyzer to the gas source;
- Clearing nasal secretions;
- Inserting a nasogastric tube;
- Setting the PEEP valve initially at 5 cmH_2_O (0.49 kPa), and gradually increasing that to a maximum of 7.5 cmH_2_O (0.68 kPa), while adjusting FiO_2_ to maintain SpO_2_ between 93 and 97%;
- Setting the flow rate according to the infant’s body size, typically from 30 L/min to 50 L/min.

An explanatory video about the H-CPAP procedure is available in the supplementary material. 

Vital signs (SpO_2_, respiratory rate, heart rate, and ECG) were continuously monitored and recorded by the nursing staff every hour initially, and every 4 h after stabilization, using individual bedside monitors. The medical staff adjusted H-CPAP parameters, if necessary, and assessed the patient at the start of treatment, within the first 60 min, after 2 h, after 6 h, and then each time a deterioration in clinical status was noted. Vital signs, H-CPAP parameters (pressure, flow, FiO_2_), and details of the nursing care and type of feeding were obtained from our online medical records. Visual telemedicine monitoring was not available on the ward. Medical and nursing staff were trained in the use of H-CPAP initially through a specific 8 h didactic course, followed by rapid-cycle, deliberate practice hands-on training on the pediatric ward during work hours on how to set up the H-CPAP machine, how to ensure good maintenance of the machine, and how to monitor its functioning. SpO_2_ was continuously and locally monitored by pulse oximetry. Tolerance to non-invasive support for respiratory failure prioritized sequential non-pharmacological strategies including using a pacifier, administrating sucrose, and engaging the present parent/familiar caregiver. If these measures did not achieve H-CPAP tolerance, the clinician would use a protocolized one-time bolus dose of dexmedetomidine (0.25 mcg/kg/dose) over 10 min for brief pharmacological sedation to facilitate tolerance of the helmet [15]. The protocolized H-CPAP weaning process started with a decrease in PEEP to 5 mmHg. If the patient tolerated this change well and maintained an oxygen saturation (SpO_2_) greater than 92% while receiving an inspired oxygen fraction (FiO_2_) between 50% and 40%, the transition to a high-flow nasal cannula (HFNC) was initiated. For the removal of H-CPAP, 2 nurses were always required. First, the oxygen device flow was turned off; then, placing fingers inside rubber collar and stretching, the H-CPAP was removed over the patient’s head. 

### 2.3. Cost-Saving Analysis

We also recorded costs of therapies using the institutional billing database. Costs are reported per day of hospitalization for each unit and presented separately in two groups based on the hospital service to which the patient was admitted (PW or PICU). 

Direct costs included medical and therapy services, diagnostics tests (hemogram, C-reactive protein), and consumables (medications, fluids, supplies, nebulization, and oxygen treatment). Indirect costs included hotel services, training costs, stationery, general institute costs, utilities, cleaning, supervision, staff costs in non-healthcare departments, and quality certifications. Costs were calculated in euros, based on the last hospital cost analysis in 2019.

Our cost analysis was conducted as follows:

- Cost A (PICU + PW) = {(days of H-CPAP treatment × Total daily cost of hospitalization in PICU) + [(total length of hospitalization − days of H-CPAP treatment) × total daily cost of hospitalization in PW]}.
- COST B (PW) = (total length of stay in PW) × (total daily cost of hospitalization in PW).
- Cost savings per individual patient = (COST A − COST B).
- Total cost saving = sum of the total cost savings per individual patient.

### 2.4. Data Analysis

We collected patient data using the electronic health records of our hospital “Bambino Gesù” and created a database for statistical analysis. We selected the following information: demographic and clinical characteristics, type of viral infection, length of hospital stay, need for transfer to the PICU, and length of stay in the PICU. Respiratory parameters were also collected over time before (baseline) and during H-CPAP therapy. The Rox index (=SpO_2_/FiO_2_/respiratory rate) [16] was also calculated before starting H-CPAP and during H-CPAP therapy. 

Data were analyzed using the statistical package SPSS version 18.0 (SPSS, Chicago, IL, USA). We performed descriptive statistics and used box plots to describe the monitoring of vital signs. Statistical differences in median values were assessed using the Mann–Whitney test and ANOVA test. We considered *p* < 0.05 to be statistically significant.

## 3. Results

Using the diagnosis code on the hospital discharge forms, we identified 130 infants diagnosed with bronchiolitis who were admitted to the emergency department of our hospital, “Bambino Gesù” in Rome, between October 2022 and February 2023.

**Figure 1:**
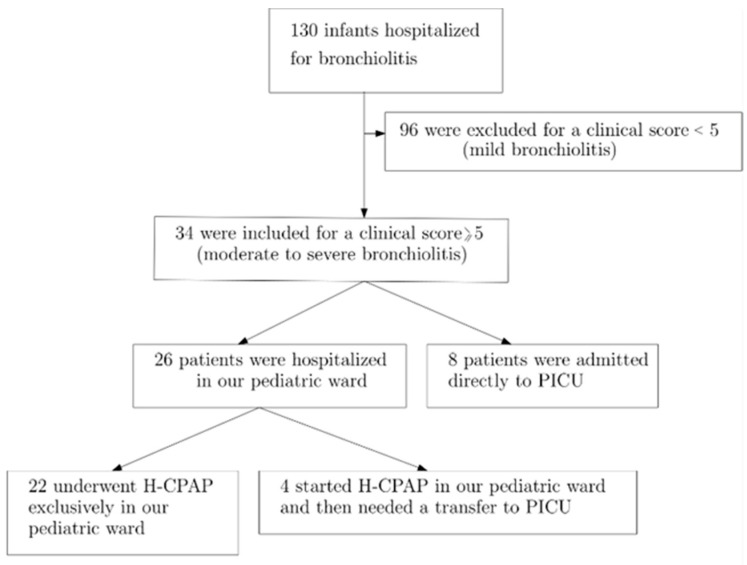
Patient flow chart.

The eligible infants were aged less than 1 year and more than 1 month, with a gestational age greater than 37 weeks, and without significant heart conditions, pulmonary diseases, or neuromuscular disorders in their past medical history. We excluded 96 infants classified as having mild bronchiolitis according to the bronchiolitis severity score [17]. We identified 34 patients as having moderate-to severe bronchiolitis (clinical bronchiolitis score ≥ 5) (Table 1): 8 of them required an immediate admission to the PICU for intensive care. The subset analysis of this study was composed of 26 patients who received H-CPAP in our pediatric ward (Figure 1). 

Among the 26 case series pediatric patients who received H-CPAP in our pediatric ward, 4 out of 26 (15%) required transfer to the PICU within the first hours of care due to clinical deterioration and the need for escalation to invasive mechanical ventilation. Of these patients, 11 (42%) were female. Additionally, 16 (61%) tested positive for RSV RNA in respiratory specimens, while none were COVID-19-positive.

The main indication for H-CPAP was severe respiratory distress (22/26, 84%), defined by the presence of pronounced intercostal retractions, grunting, and/or nasal flaring. Among the cohort, 1/26 (4.5%) started H-CPAP for apnea, and 2/26 (8%) for hypercapnia and severe respiratory distress. Prior to initiation of H-CPAP, a humidified high-flow nasal cannula (HFNC) titrated to a maximum of 2/L per minute was used, and this failed in 18/26 (69%) (Table 2). 

**Figure 2:**
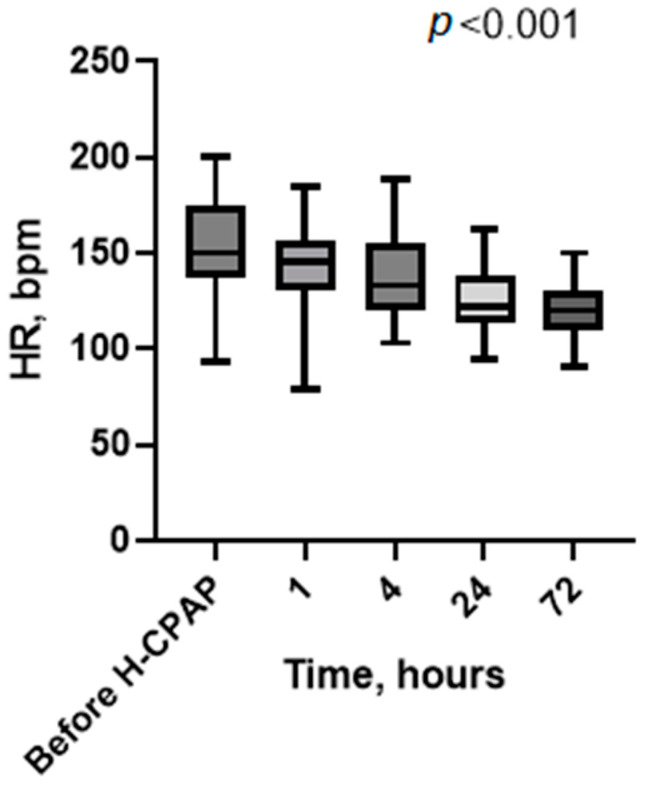
Heart rate values’ distribution at baseline (before H-CPAP) and during H-CPAP therapy. The box-whisker plots show the median (horizontal line), interquartile range (margins of box), and absolute range (vertical line). HR, heart rate (bpm); H-CPAP, Helmet Continuous Positive Airway Pressure.

**Figure 3:**
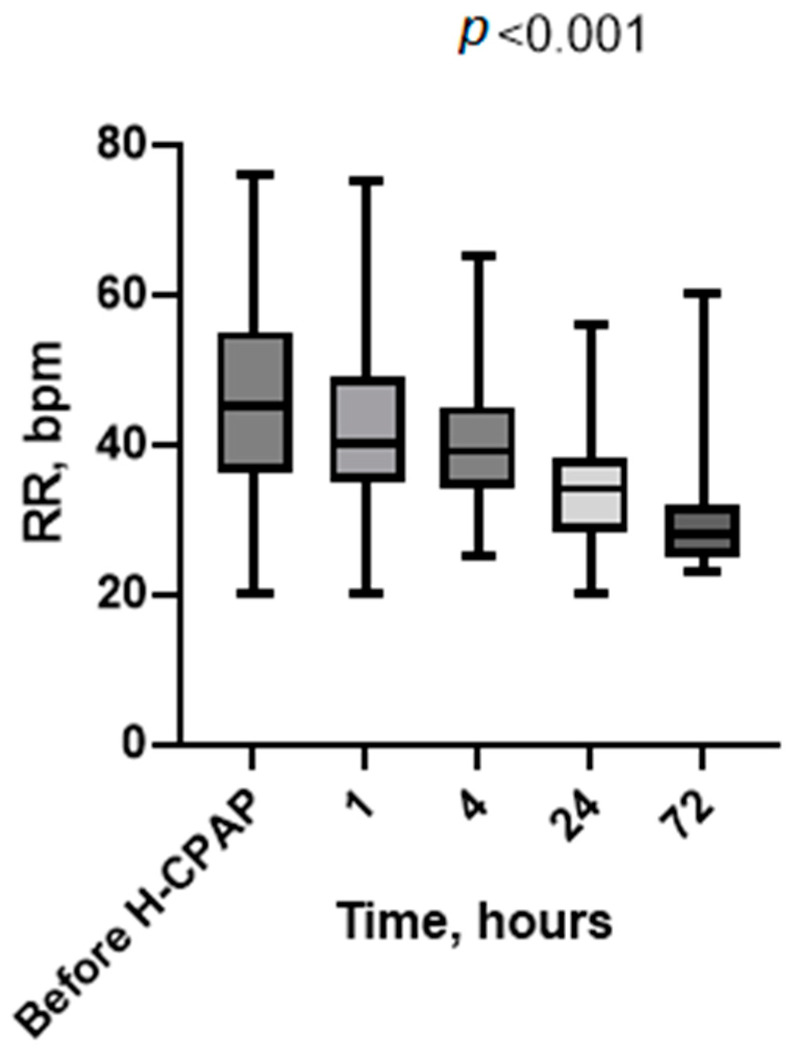
Respiratory rate values’ distribution at baseline (before H-CPAP) and during H-CPAP therapy. The box-whisker plots show the median (horizontal line), interquartile range (margins of box), and absolute range (vertical line). RR, respiratory rate in breaths per minute (bpm); H-CPAP, Helmet Continuous Positive Airway Pressure.

**Figure 4:**
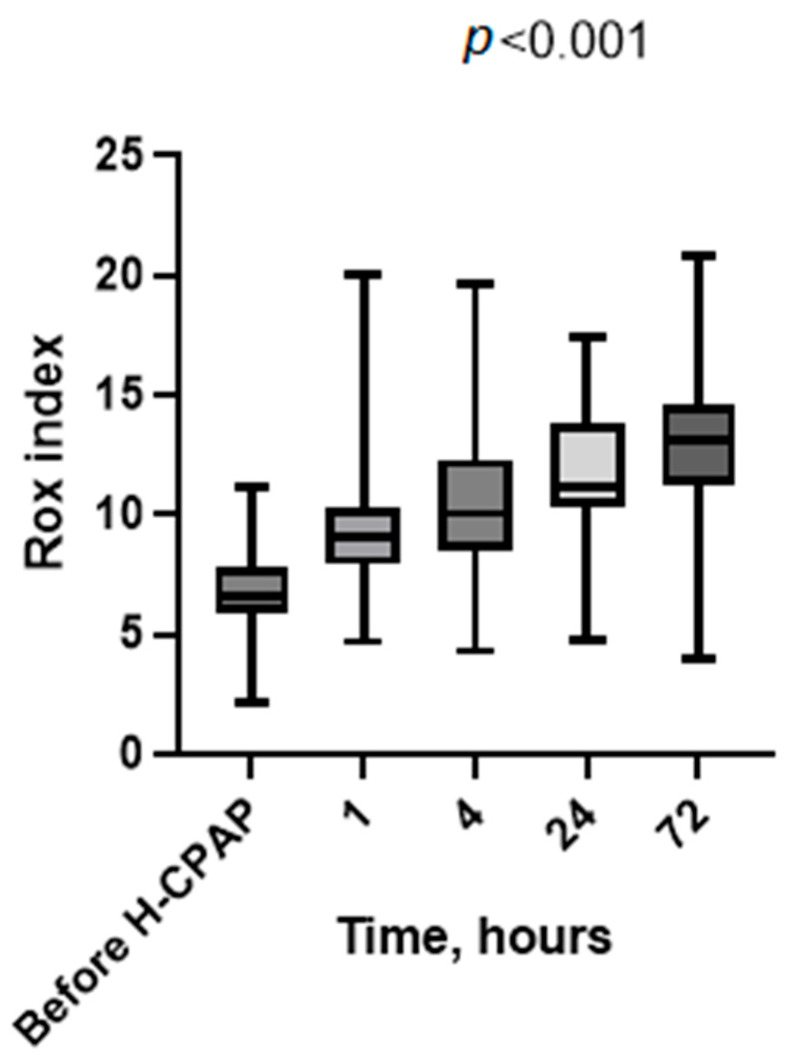
Rox index values’ distribution at baseline (before H-CPAP) and during H-CPAP therapy. The box-whisker plots show the median (horizontal line), interquartile range (margins of box), and absolute range (vertical line). RR, respiratory rate in breaths per minute (bpm); H-CPAP, Helmet Continuous Positive Airway Pressure.

Vital signs recorded at baseline (before H-CPAP) and 1, 4, 24, and 72 h showed a significative marked progressive improvement in heart rate (Figure 2), respiratory rate (Figure 3), Rox index (Figure 4), bronchiolitis clinical score, and oxygen saturation (Supplementary Table S1). 

No interface-related problems were reported, nor other side effects that would have required removal of the H-CPAP (e.g., new pneumothorax, marked gastric distension, and protracted emesis/aspiration) (Table 1). Pharmacological sedation with a single dose of dexmedetomidine was required in 15/26 patients (57%), following a failure of escalation of non-pharmacological strategies. Only two cases of relative bradycardia (minimum HR 48 bpm) related to the acute dexmedetomidine bolus were identified as adverse effects. 

In Table 3, we report the total daily hospitalization costs in the PICU and pediatric ward (PW), which amounted to EUR 2944 and EUR 492, respectively. 

We found that the median cost saving per individual patient was EUR 6687. Then, we summed the cost saved for each patient to the total number of patients who received H-CPAP exclusively in the pediatric ward during the selected epidemic season. This resulted a total cost savings of approximately EUR 147,120.

In Supplementary Table S2, we report the unit costs of the most-needed medical items and healthcare services required in patients with bronchiolitis.

## 4. Discussion

We have reported the feasibility, safety, and cost savings of using Helmet-CPAP in the pediatric ward of a tertiary care hospital with consultation and ready access to PICU staff, where providers were supported by a specialized H-CPAP curriculum and an ongoing training program for a consecutive cohort of selected patients with moderate-to-severe bronchiolitis. Additionally, we assessed the economic implications of this strategy in our unit compared to the PICU. Following the initiation of H-CPAP, we observed a progressive improvement in vital signs (heart rate, respiratory rate, SpO_2_, and Rox index) as well as a reduction in work when breathing. These findings and the timeline of improvement (2–4 h) are consistent with previous studies [5,16]. 

Prior research has demonstrated that H-CPAP can decrease the work when breathing, respiratory acidosis (pH and PCO_2_), and the length of hospital stay [18,19]. A recent review, which included 27 RCTs and a meta-analysis, compared various outcomes among H-CPAP, HFNC, and low-flow oxygen therapy (e.g., respiratory rate, heart rate, length of stay, and treatment failure) and concluded that there is currently insufficient evidence to support the superiority of CPAP over standard nasal cannula oxygen treatment or HFNC [20]. Furthermore, in a recent meta-analysis of CPAP for acute bronchiolitis in children, no differences were found between those treated with CPAP and those receiving supportive care regarding the need for mechanical ventilation [7].

In our study of H-CPAP, we did not observe complications such as severe gastric distension, emesis/aspiration, eye trauma, or pneumothorax. Helmets may theoretically provide a superior CPAP interface compared to nasal or face mask CPAP, as they reduce skin pressure and the risk of soft tissue injuries while maintaining a similar efficacy to nasal prongs in young infants with acute bronchiolitis [21,22]. Additionally, a multicenter randomized controlled trial involving 30 infants with severe acute bronchiolitis reported a lower incidence of treatment failure in the helmet group compared to the facial mask group [23]. The literature on adverse effects related to helmet interfaces in the pediatric population remains scarce, while extensive discussions have taken place regarding their use in adults during the COVID-19 pandemic [24]. Consequently, the current evidence does not favor a helmet delivery of CPAP over other methods [20].

After escalating from non-pharmacological to pharmacological sedation with dexmedetomidine, we noted only two cases of bradycardia associated with the acute bolus. Neither of these cases required intervention. The related literature supports the notion that decreasing the heart rate is a predictable physiological response, typically dose-related, to dexmedetomidine. We adhered to the recommended guidelines regarding the administration time (over 10 min) to mitigate the risk of adverse hemodynamic responses linked to rapid bolus administration of dexmedetomidine [25,26]. 

The current study suggests that the implementation of H-CPAP in the inpatient ward is a feasible option for selected cases, particularly when appropriate staff training and close access to PICU support are available [13,27]. This approach was effectively implemented, supported by specific training curricula. One important benefit of this strategy was the preservation of PICU beds during the winter season, along with potential cost savings for the public health system. Hospital admission rates in the USA and Europe are reported to be between 20 and 30 per 1000 for children under 1 year, with PICU admission rates ranging from 4% to 15% for otherwise healthy infants, leading to substantial admission costs [28]. Moreover, a recent article published in *Pediatric Pulmonology* reported on a systematic review and cost–utility analysis comparing high-flow nasal cannula (HFNC) with continuous positive airway pressure (CPAP) in the treatment of children with moderate to severe bronchiolitis in Colombia. The study concluded that CPAP was more cost-effective, offering lower costs and higher quality-adjusted life years (QALYs) compared to HFNC. Specifically, the expected annual cost per patient for CPAP was approximately USD 17,574, whereas for HFNC, it was USD 29,421 [8]. During the COVID-19 pandemic, healthcare systems faced overwhelming demands from patients requiring respiratory assistance. Consequently, H-CPAP was trialed in adults outside of ICUs with promising results, as demonstrated in several observational studies. In a study conducted in northern Italy, patients suffering from respiratory failure due to COVID-19 pneumonia were treated with H-CPAP, successfully avoiding intubation in most cases after standard oxygen therapy had failed [29]. However, experience with H-CPAP in infants with bronchiolitis outside the PICU remains limited [27].

Our study is the first conducted in Italy and one of the few international papers to report the use of H-CPAP outside of PICUs in infants with acute respiratory failure bronchiolitis, but it does have several limitations. The sample size is relatively small, and the inclusion of a control group would have strengthened the analysis. Nevertheless, extensive PICU consultation and staff training accompanied the intervention, and our experience presents a viable approach to safeguarding PICU beds and ensuring their availability for other patients during seasonal outbreaks and periods of critical care resource shortages [30].

## 5. Conclusions

H-CPAP can serve as an effective respiratory support option in selected non-intensive care settings, when providers receive comprehensive training and have access to a PICU for consultation and transfer, if needed. Future research should focus on conducting well-designed randomized controlled trials (RCTs) with larger sample sizes to further validate these findings.

## Figures and Tables

**Table 1:** Demographic characteristics and clinical features of our study cohort.

Variable	Result
Female (n) (%)	11 (42)
Male (n) (%)	15 (58)
Gestational age (wg) (median, range)	39 (30–41)
Weight at hospitalization (g) (median, range)	5100 (4200–5900)
Age at hospitalization (days) (median, range)	72 (16–345)
Length of hospitalization (days) (median, range)	5 (1–7)
Clinical score at t0 (n) (%)	
6–10	26 (100)
>10	0 (0)
Viral status (n)	
RSV	13/26
RSV-rhinovirus	2/26
RSV-influenza	1/26
RSV-metapneumovirus	2/26
RSV-negative	4/26

wg, weeks of gestation; t0, immediately before H-CPAP; RSV, respiratory syncytial virus; range refers to that between minimum and maximum values.

**Table 2:** Clinical features and complications of H-CPAP treatment in our study cohort.

Variable	Result
IMV after H-CPAP	4 (15)
HFNC before H-CPAP (n) (%)	18 (69)
Indications to start H-CPAP (n) (%)	
Severe respiratory distress	22 (84)
Hypercapnia and severe respiratory distress	2 (8)
Apnea	2 (8)
Interface tolerance (n) (%)	22 (85)
Duration of H-CPAP (hours) (median, range)	72 (4–96)
Weaning HFNC after H-CPAP (n) (%)	26 (100%)
Enteral feeding with nasogastric tube (n) (%)	26 (100)
HCPAP adverse effects	
Gastric distension (n)	0
Pneumothorax (n)	0
Bradycardia due to pharmacological sedation (n) (%)	2 (7)

IMV, invasive mechanical ventilation; HFNC, high-flow nasal cannula. Range refers to that between minimum and maximum values.

**Table 3:** Total daily cost of hospitalization in pediatric ward compared to PICU, combining direct and indirect costs.

**Direct costs**	**EUR**
In PW	376
In PICU	2398
**Indirect costs**	
In PW	116
In PICU	546
**Total daily costs of hospitalization (direct and indirect combined)**	
In PW	492
In PICU	2944

Unit cost refers to 2019 cost analysis. PW, pediatric ward. PICU, pediatric intensive care unit.

## Data Availability

Data available on request due to restrictions (privacy reasons).

## Supplementary Materials

The following supporting information can be downloaded at https://www.mdpi.com/article/10.3390/children11111273/s1: Supplementary Table S1: Parameters’ variation over time, at baseline (t0) and during H-CPAP (t1–t72). Supplementary Table S2: Unit cost of the most-needed medical items and healthcare services in patients with bronchiolitis. Video S1: H-CPAP procedure.
