# Supplementary material for: Helmet Continuous Positive Airway Pressure for Acute Bronchiolitis Respiratory Failure in a Pediatric Ward: Is It a Replicable Experience?
Source: Children (Basel). 2024 Oct 22;11(11):1273. doi: 10.3390/children11111273 (PMC11592809; doi:10.3390/children11111273)
Supplement: Supplementary file 1 [file children-11-01273-s001.zip › supplementary tables.pdf]

Supplementary tables:

Supplementary Table 1 Parameters variation over time, at baseline (t0) and during H-CPAP (t1-t72).

Parameters	t0	t1	t4	t24	t72
RR	55 (34-70)	40 (20-65)	39 (20-55)	35 (23-48)	30 (16-40)
HR	165 (116-185)	144 (106-175)	130 (100-165)	120 (90-145)	110 (80-127)
Oxygen saturation	95 (84-100)	98 (94-100)	98 (94-100)	98 (96-100)	98 (97-100)
Bronchiolitis severity score (N)					
< 5	0	0	3	17	22
5-10	26	22	19	5	0
>10	0	4	3	1	0

RR Respiratory rate. HR heart rate (bpm) (median, range) Oxygen saturation (%) (median, range). Missing data for 1 patient at t4, for 3 patients at t24 and for 4 patients at t72.

Supplementary table 2: Unit cost of the most needed medical items and health care services in patients with bronchiolitis

Medical item and health care services	**Unit cost (euro)** *a*
**Medical and therapy services**	
Doctor salary (per a 6-hour shift)	106
Nurse salary (per a 6 hour shift)	96
Emergency consultation provided by a specialist	20
**Diagnostic tests**	
Hemogram	2.06
PCR	0.88
**Medications**	
Salbutamol 1mg/ml nebulizer solution	2.3
Metilprednisolon 250 mG/5mL (IV)	6.2
Ceftriaxone 1 g (IV)	0.7
**Respiratory devices**	
Helmet-CPAP	177.3
PEEP valve	11.8
Kit Optiflow	34.9
Humidifier	46.4
Nasal cannula optiflow	36.6
Double distilled water	0.6

a Unit cost refers to 2019 cost analysis. IV intravenous
